# Chicago Pathological Society

**Published:** 1884-04

**Authors:** J. H. Tebbetts

**Affiliations:** Secretary


CHICAGO PATHOLOGICAL SOCIETY.

Regular meeting, December 10, 1883. The society was called to order by the President, Dr. Angear. The minutes of the last meeting were read and approved.

Dr. R. J. Curtiss was unanimously elected to membership. Dr. J. D. Skeer was proposed for membership by Dr. Tebbetts and Dr. Lyman.

The society then listened to the reading of a paper of unusual interest by the candidate, Dr. Skeer, giving a report of a rare case of intestinal obstruction, followed by the gift of the specimen to the society. A full abstract of this interesting report is omitted, as its publication was ordered by vote of the society.

The paper being open for discussion, Dr. Van Buren briefly mentioned a case occurring in his practice about six years ago. The patient, residing at the Sherman House, by profession a civil engineer, was taken gradually ill, having suffered for five or six days from constipation. Stercoraceous vomiting followed for three or four days almost constantly—partly liquid, sometimes solid. Ice checked, but did not stop the vomiting. The temperature continued below normal; the skin clammy; pulse not at any time accelerated, but below 70. But little nourishment could be given between the attacks of vomiting.

Dr. Van Buren could not tell what caused the obstruction, but thought it was probably not impaction. For treatment, he gave small doses of tartarized antimony, $\frac{1}{8}$ to $\frac{1}{4}$ grain, to relax the system, also opiates, which relaxed spasm, relieved pain, but did not produce much sleep.

Some stimulants were given, but no cathartics. In giving injections, he used a stomach pump with a long rectal tube, alternating large amounts of water, then air, for three days. Still the man kept vomiting. Then came a large free evacuation, and the man fully recovered. Did not consider it a case of pathological obstruction.

Dr. Clark supposed no treatment on earth would have relieved that stricture in Dr. Skeer's case, and saved the man.

Dr. Lyman considered the case one of great interest, and the paper a valuable one; the society should publish it. The specimen should be referred to a committee for microscopical examination, and thus decide whether the thickening were not of malignant origin. The case reminded him closely of one seen some years ago. A man suffering a year from dyspeptic symptoms, still showed nothing very decisive, and became somewhat emaciated, though without fever, the temperature being below the normal. Dr. Lyman's suspicion was directed to the pyloric orifice of the stomach. The patient sank lower; refused food; obstruction of tha bowel obtained, with stercoraceous vomiting for a short time, and he died. A few days before death there appeared, as indicating malignant disease, an œdematous condition of the leg. On post-mortem examination, the doctor found an annular sarcomatous infiltration of the ileo-cœcal valve, with considerable narrowing of the opening. The malignant nature of the infiltration was only detected on careful microscopic examination. Dr. Lyman thought the case reported this evening would also prove malignant; he therefore moved that the President be appointed a committee to examine the specimen and report at next meeting. Carried. On motion of Dr. Van Buren, Dr. Lyman's name was added to the committee, and on motion of Dr. Avery, that of Dr. Skeer was added to the same committee.

Dr. Lyman continuing, spoke of another interesting case, illustrating the fact mentioned by Dr. Skeer, "that a post-mortem furnished the best guide in diagnosis in such cases." A little child, born of a healthy mother in natural labor, and in good condition, had had on the second day no passage, as is quite common, but the next day seemed in pain and refused the breast. The temperature was a little elevated, and an anal examination with the oiled little finger passed in full length revealed a narrowness or constriction. An oiled catheter showed the same constriction, and engaged in it, passing to the sigmoid flexure and no farther. Fæcal matter clung to the catheter.

Dr. St. John made the same examination, and found the same condition. The bowels became distended, death occurring at the end of the week. Post-mortem examination revealed no stricture at all at any point, but the bowels were widely distended. There had been a slight passage, and the bowel was empty. The large intestine, from the middle of the transverse colon to the small intestine, showed traces of acute inflammation of an erysipelatou color, with softening, shreds of exudation outside the intestine, and muco-purulent matter within. The case was an entero-peritonitis, with paralysis from inflammation.

The only assigned cause for this in a healthy child was, perhaps, the accidental fright the mother received just before birth, in saving a two year old child from a fall down stairs. In this case stercoraceous vomiting occurred before death.

Dr. Skeer then mentioned the case of a large stout man, 55 years old, who had stercoraceous vomiting for eight days. Partially relieved, he had a second attack, caused by an active cathartic, making two weeks in all, and had a complete recovery. In this case the abdomen was much contracted.

Dr. Angear thought local inflammation of the peritonæum occurred frequently, and unsuspectedly without inflammatory reaction. A man in the army died suddenly. Post-mortem examination showed adhesive bands and old traces of peritonitis, but doubted whether this had caused his death. The doctor assisted at a post-mortem in another physician's practice, recently, of a child sick only twenty-four hours, but had had all symptoms of perforation. Was at school, and had had no doctor. Examination showed the vermiform appendix bound down to the peritonæum by old bands, and the appendix full of a mass the size of a bean. There was here a small perforation, where matter had passed into the peritoneal cavity, and yet the child had shown no grave symptoms. Dr. Angear thought there might be frequently enough inflammation to do all this mischief and yet not lay up the patient, and he not even feel ill.

Some member inquiring about the methods employed by many undertakers to preserve bodies, Dr. Lyman replied that the material used by the undertakers and injected into the abdominal cavity and intestines through long, sharp trocars, would in a few hours produce appearances of ulceration.

Rectal feeding, announced as a subject for discussion, was next before the Society, and Dr. Robison first detailed an interesting case seen recently, of a young woman 22 years old, under treatment a week for excessive vomiting; well nourished and apparently healthy, no known cause could be assigned, unless from nervous prostration. All the usual remedies having been employed at the time he was consulted, he simply gave morphia, oxalate of cerium and bismuth, also lime water, but still without benefit, and the patient almost in collapse. The prostration was extreme, hence stopped all medication and gave rectal alimentation, injections of milk, brandy and water, also beef peptonoids, one tablespoonful in four ounces of water at each injection. In a few hours the vomiting ceased, strength began to return, and the patient greatly improved for five days, during which time no medicine or ordinary nourishment was administered; and the doctor thought complete recovery would have followed had not the friends, on their own responsibility, administered castor oil, starting anew the vomiting, and resulting in death in about six hours.

Dr. Van Buren had never placed much reliance upon rectal feeding. This case just cited would serve to create some confidence in the subject. For purposes of stimulation, he considered rectal administration of quinine, brandy, etc., very valuable, as in collapse of cholera.

Dr. Robison then spoke of a recent case in the practice of Dr. Ross, of nervous prostration, in which feeding by the mouth was stopped altogether and rectal feeding continued seven days, when he lost sight of the case, but had subsequent good report of the patient. Dr. Robison was unable to state the form of food used.

Dr. Skeer mentioned a case of gastric ulcer, with the following treatment. For three weeks the stomach was supplied only with white of egg, and rectal feeding employed, consisting of eight ounces of beef tea, and five grains quinine, three times a day. There was no fever and no great thirst, but during the last week considerable hunger between "meals," and the patient was strong enough to have gone down stairs if allowed. The doctor thought life could be prolonged and sustained indefinitely by nutrient enemata. In using quinine, it was well never to use muriatic acid to dissolve the drug, as it will start up an obstinate diarrhœa, as in the case of President Garfield. The bisulphate will dissolve readily, and is almost immediately absorbed. He did not use milk in the rectum, for though the salts and water are soon absorbed, the curd remains as an irritating material.

A visitor then mentioned a case under the care of Dr. Gunn, of a boy with contracted œsophagus caused by swallowing hot lye ; rectal feeding had been employed exclusively for a year, no food having been taken into the stomach during that time.

Dr. Lyman had used broth and whey in rectal feeding before the day of beef peptonoids, which ought theoretically to be of great value.

Dr. Angear then in a few words mentioned the conflict in opinion and reasoning between physiologists and non-physiologists in the matter of rectal feeding. The rectal mucous membrane cannot digest albumen, nor change starch into sugar. These substances in tbe rectum only putrefy, show vibricæ, and decompose. Experiments made in Germany on patients operated upon for hernia, and having an artificial anues, show that the fluids of the large intestine will digest nothing; food placed there, at the end of a month had undergone no digestive changes, and the sharp edges of fragments of albumen were not rounded off.

Casein, fibrin, etc., are not absorbed, for digestion must first occur.

Fluids are absorbed slowly in the large intestine, about seven ounces in twenty-four hours.

Many do not stop to think how long a man will live absolutely without food;—sixty-three days is the longest time reported. Rectal feeding continued forty days is considered noteworthy, but the patient might live twenty days longer on nothing but water.

The doctor thought peptones produced irritation of the rectum, and inflammation enough to stop absorption, and destroy what little hope there may be left. In cases where rectal feeding has been honestly employed for long periods, food must have passed through the ileo-cœcal valve into the small intestine, and to bring this about, he advised using a long tube, small amounts of food frequently repeated, friction over the abdomen, etc.

Members present, about twenty, and several visitors.

The society on motion adjourned.

J. H. TEBBETTS, Secretary.

